# Characteristics of the Activity-Affect Association in Inactive People: An Ambulatory Assessment Study in Daily Life

**DOI:** 10.3389/fpsyg.2013.00163

**Published:** 2013-04-08

**Authors:** Birte von Haaren, Simone Nadine Loeffler, Sascha Haertel, Panagiota Anastasopoulou, Juergen Stumpp, Stefan Hey, Klaus Boes

**Affiliations:** ^1^Research Group hiper.campus, House of Competence, Karlsruhe Institute of TechnologyKarlsruhe, Germany; ^2^Institute of Sport and Sports Science, Karlsruhe Institute of TechnologyKarlsruhe, Germany

**Keywords:** ambulatory assessment, affect, daily life, inactive, physical activity

## Abstract

Acute and regular exercise as well as physical activity (PA) is related to well-being and positive affect. Recent studies have shown that even daily, unstructured physical activities increase positive affect. However, the attempt to achieve adherence to PA or exercise in inactive people through public health interventions has often been unsuccessful. Most studies analyzing the activity-affect association in daily life, did not report participants’ habitual activity behavior. Thus, samples included active and inactive people, but they did not necessarily exhibit the same affective reactions to PA in daily life. Therefore the present study investigated whether the association between PA and subsequent affective state in daily life can also be observed in inactive individuals. We conducted a pilot study with 29 inactive university students (mean age 21.3 ± 1.7 years) using the method of ambulatory assessment. Affect was assessed *via* electronic diary and PA was measured with accelerometers. Participants had to rate affect every 2 h on a six item bipolar scale reflecting the three basic mood dimensions energetic arousal, valence, and calmness. We calculated activity intensity level [mean Metabolic Equivalent (MET) value] and the amount of time spent in light activity over the last 15 min before every diary prompt and conducted within-subject correlations. We did not find significant associations between activity intensity and the three mood dimensions. Due to the high variability in within-subject correlations we conclude that not all inactive people show the same affective reactions to PA in daily life. Analyzing the PA-affect association of inactive people was difficult due to little variance and distribution of the assessed variables. Interactive assessment and randomized controlled trials might help solving these problems. Future studies should examine characteristics of affective responses of inactive people to PA in daily life. General assumptions considering the relation between affect and PA might not be suitable for this target group.

## Introduction

The relationship between physical activity (PA) and different affective states has been studied for decades. Early studies focused on the association between acute and regular exercise on specific negative affective states such as depression and anxiety (Ekkekakis and Petruzzello, 1999). There is evidence that exercise reduces self-reported negative affective states, namely anxiety and depression (Arent et al., 2000; Landers and Arent, 2001; Wipfli et al., 2008; Rethorst et al., 2009). Later studies integrated a rather dimensional approach of affect including positive and negative affective states.

Reed and Ones (2006) published a review about the effects of acute exercise on positive activated affect. People with lower pre-exercise affect values had higher increases in positive activated affect. Low intensity as well as moderate and vigorous exercise increased post exercise affect, with the highest observed increase after low intensity exercise. The increase of positive activated affect in response to low and moderate doses of exercise seems to be generalizable, with low intensity having the highest effect sizes (*d* = 0.57). There is more variability in affective responses to high intensity exercise. Both short and long bouts of acute exercise can induce affective improvements; however exercises lasting longer than 75 min seem to decrease positive affect. Even short bouts of brisk walking can increase activation and positive affect (Ekkekakis et al., 2000). Exercise intensity seems to have an important impact on post exercise affect and future adherence to exercise. Based on several study results, the dual mode theory was developed to show that valence declines beyond the aerobic-anaerobic transition (Bixby et al., 2001; Ekkekakis and Petruzzello, 2002; Ekkekakis and Acevedo, 2006). The authors claimed that affective states have to be assessed more frequently during exercise because affective states during exercise may explain the variability in post exercise affective states as response to high intensity exercise. Their assumption was confirmed by recent studies showing that especially inactive people show higher affective states if exercise intensity is moderate. In addition, it was recently shown that self-selection of activity intensity may help to identify the activity intensity that fits best for an individual to increase affect (Ekkekakis et al., 2011).

Considering the effects of regular exercise on positive activated affect, research draws a similar picture, regular aerobic exercise increases positive affect (Berger, 2000; Ekkekakis et al., 2000; Reed and Buck, 2009). Participants with lower baseline positive affect values had larger increases in positive affect. The highest effects were found for high and low intensity exercise programs. In contrast to the variance of affective reactions to acute sessions, repeated high intensity exercise seems to, maybe due to physiological and psychological adaptations, reach a similar level of affective changes as low intensity exercise (Reed and Buck, 2009). Additionally, the positive affective changes observed in regular exercise programs seem to be independent of training response and fitness changes (Ekkekakis et al., 2000) and the magnitude of effects was similar in acute and regular exercise (Reed and Ones, 2006; Reed and Buck, 2009).

While earlier studies focused on the potential of acute and regular exercise to improve affect, PA in daily life has become an important issue. Current activity guidelines promote daily PA and accumulated short bouts (duration of 10 min) of daily PA to be health effective (Haskell et al., 2007). As a consequence interventions recently started to include the promotion of single short bouts of activity to become more active. Thus, a growing number of studies try to examine whether PA in daily life also has the potential to improve positive affect (Schwerdtfeger et al., 2008; Kanning and Schlicht, 2010; Hyde et al., 2011; Poole et al., 2011; Kanning et al., 2012; Wichers et al., 2012).

Hyde et al. (2011) showed that people who were more physically active in general had higher pleasant activated feelings than less active people. Moreover, higher levels of pleasant activated feelings arose on days people were more active than typical for them. Kanning and Schlicht (2010) examined 13 older adults and revealed that subjectively reported activities in daily life increased energetic arousal and calmness. The authors assumed that PA is able to modify mood if there is a low baseline level but is not able to induce changes if mood state is already high.

To assess the relationship between affective states and PA in daily life, ambulatory assessment studies seem to be an appropriate method. Earlier studies mainly used retrospective self-report measures of affect and PA which are vulnerable to recall bias (Ebner-Priemer and Trull, 2009). Ambulatory assessment involves repeated respectively continuous sampling of current behavior and emotional reactions in real time. Thus, this approach allows the capturing of individual variability in affective responses to physical activities. Thereby it provides the potential to identify responders and non-responders that cannot be detected by means of the group aggregate considering the fact that one individual does not always react the same way (Backhouse et al., 2007; Shiffman et al., 2008). Additionally subjective self-reports of physical activities tend to overestimate activity and correlations between objective and subjective measures are low to moderate (Prince et al., 2008; Bussmann et al., 2009). Ambulatory assessment has the potential to measure PA occurring in daily life quite accurately *via* accelerometers. Furthermore self-reported affective states as well as contextual information can be assessed “*in situ*” (Fahrenberg et al., 2007; Bussmann et al., 2009).

Kanning et al. (2012) did an ambulatory assessment study with 44 university students. They assessed PA continuously *via* accelerometry, affective states, and the relative autonomy index were conducted with electronic diaries every 45 min for 1 day. They replicated the findings of Kanning and Schlicht (2010) for the valence and energetic arousal dimension being higher due to higher PA. In contrast to Kanning and Schlicht (2010) the calmness dimension was negatively correlated with PA. In addition, autonomous regulation moderated the PA-affect association.

Another ambulatory monitoring study in 24 healthy participants (aged 18–73 years) assessed the relation between PA and mood in daily life. Schwerdtfeger et al. (2008) recorded PA for 1 day and assessed positive activated and negative affect every hour *via* PDAs. They showed significant effects of PA (5, 10, and 15 min before PDA prompt) on positive activated affect, but not on negative affect. Even low intensity walks predicted higher positive activated affect.

Wichers et al. (2012) examined the effects of PA on positive and negative affective states in a large sample of 504 people. PA and affect were both assessed subjectively with electronic diaries. Participants had to state the current context (activity, location, social contact) and affect every 90 min on five consecutive days. Higher activity levels indicated higher subsequent levels of positive affect, but not of negative affect.

Affect is assumed to be an important motivator for continuing and maintaining PA (Dishman, 1990). Despite the postulated “feel better effect” of acute and regular exercise as well as PA in daily life, drop outs in exercise interventions are high and adherence to regular exercise and PA is low. More than half of our population is inactive (Centers for Disease Control and Prevention, 2008). For example, less than half of the US population meets the current PA guidelines (Haskell et al., 2007) and the problem can be seen worldwide (Bauman et al., 2009). Sedentary behavior is an increasing health risk in our society (Owen et al., 2010b).

Latest research showed that people can suffer from metabolic and cardiovascular health risk factors or diseases if they have prolonged sedentary episodes, even if they achieve the activity guidelines (Hamilton et al., 2004; Warren et al., 2010; Koster et al., 2012).

As a consequence, one of the main public health goals currently is the interruption of these long sitting times through performance of light activities. Light intensity activity was beneficially associated with resources against health risks (Hamilton et al., 2007). Light activities contribute to overall energy expenditure, but do not count for achieving the activity guidelines yet. Reducing prolonged sitting time through “sedentary breaks” calls growing attention as potential “easy to introduce intervention” in everyday life and has been shown to have beneficial impact on health (Healy et al., 2008; Brown et al., 2009; Owen et al., 2010a). Based on the evidence that not only exercise but also PA in daily life is health preventive, the question whether PA in daily life also has the potential to increase affect was studied more intensively. The importance to reduce long sitting times in addition to be active raises the question whether light activities performed to interrupt prolonged sitting offer the potential to increase affect in daily life.

There is growing evidence in low intensity activity being effective for health and mental well-being (Ekkekakis et al., 2000, 2008; Hamilton et al., 2004; Camhi et al., 2011). Inactive people may also profit from low intensity exercise (Bixby and Lochbaum, 2006; Carels et al., 2006). Daley and Welch (2003) found that sedentary inactive people had the highest affect ratings during low intensity exercise.

Unstructured PA in daily life includes a lot of low intensity physical activities such as walking from one place to another. There is consensus of different scientific fields that affect plays a central role in decision making (Baumeister et al., 2007) and this also seems to be suitable for the decision to engage in PA and exercise (Carels et al., 2006). Based on a finding of Simonen et al. (2003), Ekkekakis et al. (2005, p. 484) interestingly assumed that there is a “genetically determined pleasure-based mechanism that has evolved to reward and promote PA.” Moderate activities (intensities below the lactate threshold) made up most of people’s time for hundreds of years to ensure surviving (Ekkekakis et al., 2005). Along with the industrialization, the increasing sedentariness induced obesity and a reduced fitness level, too. Ekkekakis et al. (2005) assume that, in combination with factors as muscular and skeletal aches, as well as cognitive factors and physical ineffectiveness, affective responses to moderate intensity have changed negatively. Thus, it is possible that the pleasure-based mechanism to reward and promote PA is light but not moderate activity for inactive people.

If low intensity PA increases positive affect, sedentary people might choose such activities to increase PA levels in daily life. Sedentary persons might perceive low intensity activities as less aversive compared to high intensity activities (Daley and Welch, 2003). Interventions to promote low intensity activity in daily life such as enhancing the accumulation of short bouts of activity or reducing longer periods of sedentary behavior by sedentary breaks may induce different affective reactions. Schwerdtfeger et al. (2008) found that the higher the body mass index (BMI) of participants, the higher was the association between PA in daily life and energetic arousal/positive affect. They assumed that obese people may be able to influence more positively energetic arousal, which is generally lower in this population. The study did not take into account habitual PA as a moderator of the relation between affect and PA in daily life. Thus one cannot conclude that the results found in obese people can automatically be transferred to sedentary people but there is a close link between sedentariness and obesity.

In conclusion, research shows that acute and regular exercise has the potential to enhance positive affect and these effects partly apply for sedentary people (Bixby and Lochbaum, 2006; Carels et al., 2006).

The exercise intensity seems to be an important issue considering adherence and dropout rates from exercise programs of sedentary people. Studies show that sedentary people prefer exercises of lower intensity compared to active people and exercise of lower intensity can improve affective states (Parfitt et al., 2006; Ekkekakis et al., 2011).

In addition, ambulatory assessment studies showed that unstructured PA in daily life can increase affect. These studies account for the importance of inter-individual differences and perceptions for activities inducing increases in affect. In addition an individual’s affect in response to PA in daily life can be captured in many different situations. High positive affective states after PA in daily life were observed especially for light and moderate activities. Out of the studies that analyzed the association between unstructured PA in daily life and affective states, none examined sedentary people or controlled fitness and habitual PA level. As habitual exercise participation can mediate affective responses to acute bouts of exercise (Hallgren et al., 2010), this may also be appropriate for habitual PA.

Unstructured PA of moderate and light intensity in daily life may be a better opportunity to achieve and maintain higher activity levels in inactive people than exercise. In addition recent research shows that reducing prolonged sedentary episodes through performance of light activities is important besides achieving current activity guidelines.

Thus this pilot study aimed at providing new insights in the PA-affect association of inactive people in daily life. Because we were interested in affective states after PA in different situations of an individual in real life, we used the method of ambulatory assessment.

We hypothesized that PA in daily life was associated with subsequent affective state in inactive people. Thus we analyzed how PA was associated with subsequent affective state.

In a secondary analysis, we addressed a possible association between interrupted sedentariness and affective states. To our best knowledge no study has yet examined whether breaks in prolonged sedentary episodes are associated with affective states in young inactive adults in daily life.

## Materials and Methods

### Participants

Twenty-nine students (mean age: 21.3 ± 1.7 years; mean BMI: 24.5 ± 4.4) of the electrical engineering and information technology department (3rd and 5th semester) were recruited at a German University. They were informed about study goals and written consent was obtained according to the guidelines set for by our institution. All participants were inactive (being defined as exercising once a week or less) and had a mean relative VO_2_ max of 40.9 (±5.6) ml/min/kg measured *via* gas analysis during a progressive treadmill test.

### Physical activity

Physical activity was assessed with the Move II sensor (movisens GmbH, Karlsruhe, Germany) placed to the chest. Move II consists of a triaxial acceleration sensor (adxl345, Analog Devices) with a range of ±8 g, 64 Hz sampling frequency and 12 bit resolution. The measuring unit has an additional air pressure sensor (BMP085, Bosch GmbH) with a sampling frequency of 8 Hz and a resolution of 0.03hPa (corresponding to 15 cm at sea level). The recorded raw data was saved on a SD card and was transferred to a computer for further analysis *via* a USB 2.0 interface.

Based on the hypothesis, we operationalized PA in two ways. First we calculated mean activity intensity and energy expenditure expressed by the MET value. Using MET values is an established method to estimate and classify the energy cost of human PA. Activity intensity is displayed on the basis of their energy cost as multiple of 1 MET, which is defined as the energy cost of a person at rest (Ainsworth et al., 2011). Activities ranging from 1 to 1.5 MET are defined as sedentary, from 1.6 to 2.9 MET as light, 3–5.9 as moderate, and ≥6 as vigorous (Ainsworth et al., 2011). Second, we calculated the minutes spent in the light activity category to analyze whether the proportion of time spent in light activities during the activity episode was associated with subsequent affect.

In the first step the activity was classified in intervals of 4 s. For further explanation of the activity recognition process see the publication of Anastasopoulou et al. (2012). The classification algorithm differentiated between the following seven activities: lying, rest (sitting/standing), cycling, uphill, downhill, level walking, and jogging. Based on the detected activity class, the appropriate activity-dependent EE model was selected and the EE was estimated. Input for the EE estimation models were the acceleration magnitude, the altitude change, and some subject related parameters (sex, age, height, and weight). MET was calculated as the ratio of the associated metabolic rate for the specific activity divided by the resting metabolic rate (RMR) (for details, see FAO/WHO/UNU, 1985).

### Affective state

For the assessment of affective states, My Experience movisens Edition (movisens GmbH, Karlsruhe, Germany) was used to install the mood scale on the PDAs (personal digital assistant; HTC Touch Diamond 2) and to program time stamp and intervals between the diary prompts. We used a six item short scale (Wilhelm and Schoebi, 2007) which measures the three basic mood dimensions with two bipolar items for each dimension: energetic arousal (E: tired-awake; full of energy-without energy); valence (V: content-discontent; unwell-well) and calmness (C: agitated-calm; relaxed-tense). The scale was developed based on the Multidimensional Mood Questionnaire (Steyer et al., 1997) and validated especially for momentary assessment of mood in daily life (Wilhelm and Schoebi, 2007). Participants rated their current mood based on the statement “At the moment, I feel…” on a seven-point bipolar rating scale with two opposed adjectives as endpoints (0–6). Prior to analyses three items were reversely coded to ensure that higher scores indicated higher values of E, V, and C. Subscale scores for each dimension ranged from 0 (low value) to 12 (high value).

### Procedure

The measurement equipment was handed out to the participants in an introductory session at the university 1 day prior to the start of the assessment period. PA was objectively conducted with the Move II accelerometer (movisens GmbH, Karlsruhe, Germany) over 12 h on 2 days (10 a.m.–10 p.m.). Participants had to record the duration during which they did not wear the device. Ratings of affective states and activities were prompted *via* PDAs. Participants were alerted by the vibrating signal of the PDA approximately every 2 h between 12 a.m. and 10 p.m. with a random component to avoid subjects waiting for the signal. Subjects had to answer the prompts within 15 min, and if they missed a signal, they were reminded by two further signals after 5 and 10 min. PDA morning entries (10 a.m.) were excluded from the analyses because the accelerometer registration of PA started at 10 a.m., and analyzing the previous 15 min of the 10 a.m. PDA entry was, therefore, not possible. Additionally, the variable waking time of subjects caused numerous 10:00 a.m. prompts to be missed. PDA and accelerometer time were synchronized by the initialization of the computer.

### Data analyses

PDA prompts corresponding to non-wearing times of the activity sensor were excluded from the analyses. MET values were averaged across data points for each minute in a 12 h period for both days (10:00 a.m.–10:00 p.m.).

To analyze the within-subject association between preceding PA and mood, the average PA intensity was calculated over the preceding 15 min before the PDA prompt. Based on recent studies, a 15 min interval was chosen to include as much activity as possible but within a time frame that ensures that increased affect after activity would still be visible (Ekkekakis et al., 2000; Schwerdtfeger et al., 2008). The mean MET level was calculated by averaging MET values (per minute) of this time interval. To investigate the potential of light activities to increase affective states, we calculated minutes spent in light activities (1.6–2.9 MET). This level is the most performed activity of sedentary people. Thus, one might get more intervals of light activity compared to moderate or vigorous intervals, especially in an inactive sample. We summed up minutes spent in light activity over the 15 min interval and did within-subject correlations for the three affect dimensions with light activity.

For the secondary analysis we calculated the number of sedentary breaks to reflect interruptions of at least 1 min in sedentary time. Thus, one sedentary break reflected an interruption of sedentary behavior (≤1.5 MET) of at least 1 min. To calculate breaks of sedentary periods, we used intervals of 30 min length.

For this pilot study within-subject correlations of the activity and affect variables were assessed to show the range of within-subject correlations in the sample. Due to the small sample size (*n* = 29) and the lack of variance in activity, as well as the distribution of the affect variables, we decided to take a more robust statistical method instead of using multilevel analyses. Therefore we calculated the within-subject correlations between activity variables (mean MET, minutes spent in light activity and sedentary breaks) and the three dimensions (valence, energetic arousal, and calmness) of affect. To calculate the mean correlation coefficient, we did Fisher’s z-transformation to convert Pearson’s *r* values into normally distributed *z* values. The mean correlation coefficient was transformed back *via* inverse fisher’s z. Significance (p) was tested *via* *t* distribution (Bortz and Schuster, 2010).

## Results

### Association between activity intensity/minutes spent in light activity and affect

Three hundred thirty-four data points for activity variables and corresponding affective state variables were available. Across all 15 min intervals, participants had a mean activity intensity level of 1.44 (±0.42) MET. Participants spent on average 1.62 (±2.46) min in light activity across all 15 min intervals. Across all 15 min intervals, participants spent 86% in sedentary behavior, 10.4% in light activities and 3.6% in moderate to vigorous activity. The activity spent during the 15 min intervals was significantly associated to overall measured PA (2 days) for both mean MET level (*r* = 0.724; *p* = < 0.001) and minutes spent in light activity (*r* = 0.710; *p* = 0.00). Mean affective states were 7.44 (±2.41) for energetic arousal, 8.78 (±1.94) for valence, and 8.74 (±2.2) for calmness. Participants rated the two measurement days as either quite (3) or predominantly (4) typical (on a scale ranging from 1 not at all to 5 absolutely).

**Figure 1:**
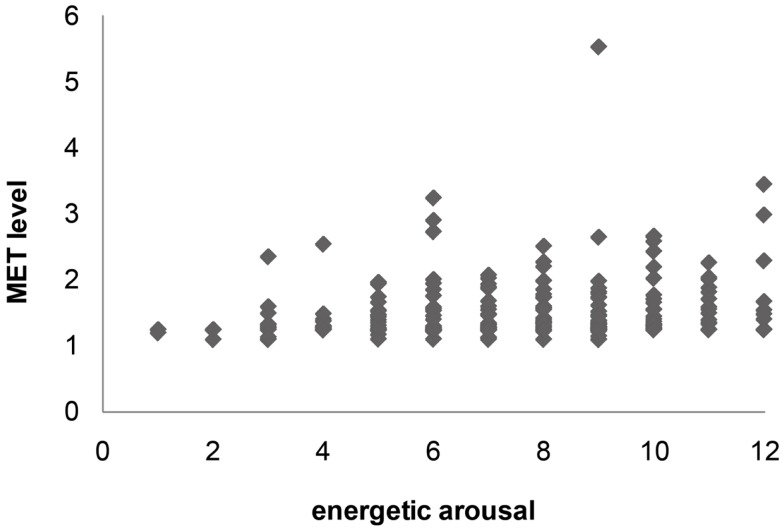
**Scatter plot of E (energetic arousal) score and activity intensity (mean MET level during 15 min interval)**.

By calculation of 90th and 10th percentiles, we identified that 80% of the data points of E were located between the affect scale scores of 4 and 10, and 80% of V and C were located between the scores 6 and 11 (Table 1). The calculation of 10th and 90th percentiles for both mean MET level (10th = 1.25; 90th = 1.94) and minutes spent in light activity (10th = 0; 90th = 5) showed that most of the time was spent in an intensity reflecting primarily sedentary behavior (see Table 1; Figure 1).

**Table 1:** **Means and standard deviations of the activity variables mean MET, minutes spent in sedentary, light, moderate, and vigorous activity (across all 15 min intervals); sedentary breaks (across all 30 min interval) and affect variables (across all diary prompts); 90th and 10th percentile of each variable**.

	Mean (SD)	10th percentile	90th percentile
Mean MET	1.45 (±0.42)	1.25	1.94
Min sedentary	13.39 (±3.75)	8	15
Min light	1.63 (±2.46	0	5
Min moderate	0.51 (±1.38)	0	2
Min vigorous	0.04 (±0.51)	0	0
Sedentary breaks	5.05 (±5.8)	0	14
Energetic arousal (E)	7.44 (±2.41)	4	10
Valence (V)	8.78 (±1.94)	6	11
Calmness (C)	8.74 (±2.2)	6	11

None of the affect variables, E, V, and C, showed significant associations to the activity intensity level precedent to the affect assessment (Table 2), with energetic arousal showing the highest mean correlation coefficient for both activity variables, mean MET level (*r* = 0.17; *p* = 0.82) and minutes spent in light activity (*r* = 0.13; *p* = 0.75). The within-person correlations between energetic arousal (E) and mean MET level ranged from *r* = −0. 42 to 0.89. To illustrate the number of persons having low, high negative, or high positive correlation coefficients, the results of the correlations were categorized [1 = 0.3–0.99; 0 = −0.29 to 0.29; −1 = −0.3 to −0.99]. Sixteen subjects had a correlation coefficient between 0.29 and −0.29, 10 subjects had a correlation coefficient of 0.3–0.99 and three subjects fell into category −1 (−0.3 to −0.99). The within-person correlations for the association between minutes spent in light activity and E ranged from *r* = −0.42 to 0.93 (see Table 2). Participants rated higher as well as lower energetic arousal when they spent little time in light activity. Low ratings of E together with high numbers of minutes spent in light activity did not occur.

**Table 2:** **Correlation coefficient (*r*) of within-subject correlation (*n* = 29; df = 27) between mean activity intensity (mean MET during 15 min interval) and E (energetic arousal), C (calmness), and V (valence); amount of minutes spent in light activity (during 15 min interval) and E, C, V; sedentary breaks (during 30 min interval) and *E*, *C*, *V*; range of within-subject correlations; significance (*p*); effect sizes (*r*^2^)**.

	*r*	Range	*T*	*p*	*r*^2^
mean MET_E	0.17	−0.42–0.88	0.92	0.82	0.03
mean MET_C	−0.09	−0.90–0.52	−0.45	0.33	0.01
mean MET_V	−0.03	−0.97–0.67	−0.13	0.45	0.00
min_light_E	0.13	−0.42–0.93	0.69	0.75	0.02
min_light_C	−0.08	−0.94–0.35	−0.44	0.33	0.01
min_light_V	0.08	−0.93–0.94	0.40	0.65	0.01
sed_breaks_E	0.20	−0.57–0.82	1.04	0.85	0.04
sed_breaks_C	−0.08	−0.81–0.70	−0.42	0.34	0.01
sed_breaks_V	0.07	−0.83–0.85	0.38	0.65	0.00

**Figure 2:**
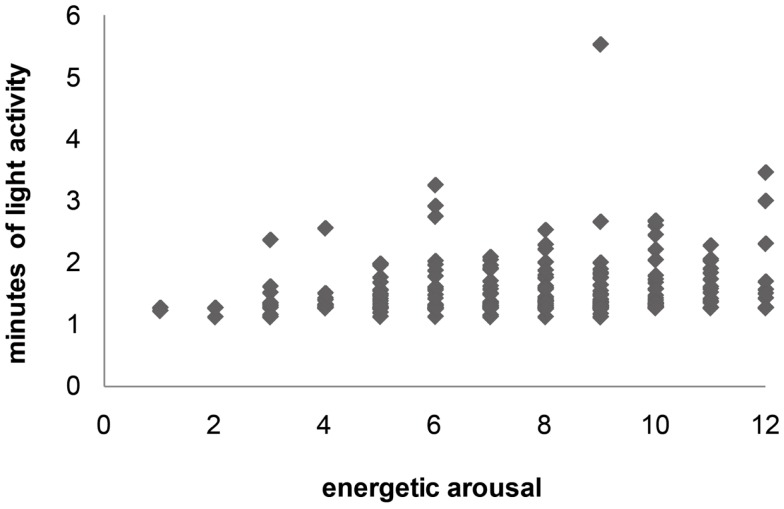
**Scatter plot of E (energetic arousal) score and amount of minutes spent in light activity (during 15 min interval)**.

The results of the within-person correlations of the two other mood dimensions, valence and calmness, showed even lower correlation coefficients (Table 2). Within-subject correlations between valence and mean MET level ranged from −0.97 to 0.67, (min light: *r* = −0.93–0.94). The within-subject correlations between calmness and mean MET ranged from −0.90 to 0.52 (min light *r* = −0.94–0.35). Participants rated higher as well as lower affective scores (valence, calmness) when they spent little time in light activity. Low ratings of affective states together with high numbers of minutes spent in light activity did not occur (see Figure 2).

### Secondary analysis: Association between sedentary breaks and affect

**Figure 3:**
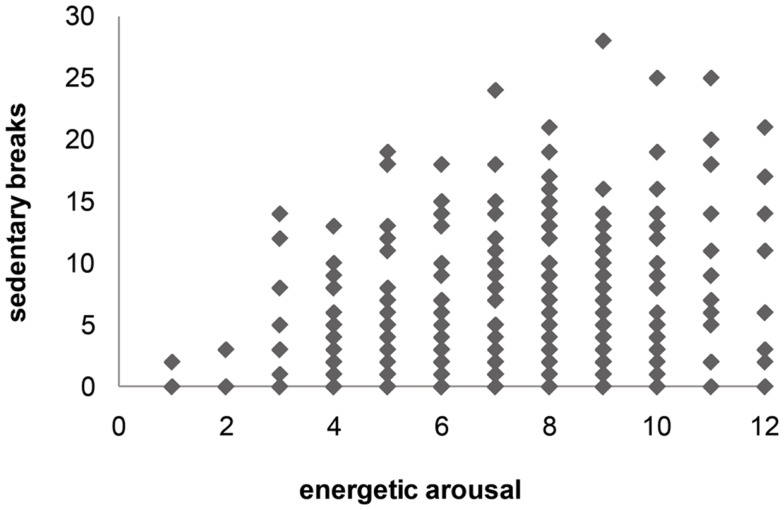
**Scatter plot of E (energetic arousal) score and sedentary breaks (during 30 min interval)**.

Across all 30 min intervals, the mean number of sedentary breaks was 5.1 (±5.8). The scatter plot shows that few as well as lots of sedentary breaks during the 30 min interval induced higher as well as lower arousal states. Participants did not have low arousal states and a high number of sedentary breaks at the same time (see Figure 3).

Again energetic arousal showed the highest correlation with sedentary breaks (*r* = 0.2; *p* = 0.85) with a range of within-subject correlations from −0.57 to 0.82, but did not reach significance. Valence and calmness were not related to sedentary breaks (Table 2).

## Discussion

In the present pilot study we investigated whether the PA intensity level of inactive young adults was associated with affective states in daily life. Moreover, we were interested whether affective states of inactive young adults changed in response to the amount of time spent in light activities. Our results are not in direct agreement with earlier studies demonstrating a relationship between PA and positive affect in daily life (Schwerdtfeger et al., 2008; Kanning and Schlicht, 2010; Hyde et al., 2011; Kanning et al., 2012; Wichers et al., 2012). We found small and statistically insignificant associations between energetic arousal and activity intensity level as well as low intensity activity. The two other affect dimensions, valence and calmness were not related to PA.

Despite the variety of variables used to display activity (active versus inactive episodes, number of counts, milligram) and the fact that other studies did not control habitual activity or fitness level of participants (or did not report it), it seems adequate to assume that our sample was more inactive compared to samples of former studies. Kanning and Schlicht (2010) registered 28% of active episodes and 72% of inactive episodes, compared to 86% inactive and 14% active (mainly light activities) episodes in this study. Kanning et al. (2012) reported 77.3 mg/min compared to the mean MET level of 1.44 MET in the present study (reflecting the sedentary behavior category).

Compared to earlier studies, we extended our analysis to 2 days. Thus, we reduced the risk for picking an untypical day, but reduced the chance to capture activity episodes by programing PDA prompts every 2 h (compared to every hour).

The distribution of the valence and calmness dimension variables was shifted to high positive affective state scores. It is possible, that for people having general high mood scores, PA is less effective in altering mood. Kanning and Schlicht (2010) as well as Gauvin et al. (1996) discuss ceiling effects as a possible explanation and studies show stronger improvement in affect if the baseline level is low (Reed and Ones, 2006). The constant high valence and calmness levels of subjects in this study may partially explain the lack of finding an activity-affect relation.

The results of the present pilot study highlight the difficulty of analyzing the relation between affect and PA of inactive people. It is difficult to relate affective states to active episodes if they hardly appear during the day (Ebner-Priemer et al., 2013). We anticipated the difficulty to capture a lot of vigorous and moderate activity episodes, but we did not expect that even light activity episodes would hardly occur in the present study. Of the 334 data points, only 60 had a mean MET level >1.6. The calculation of the 10th and 90th percentiles illustrated the immense lack of variance of the activity data, thus the data was insufficient to detect associations.

To solve the problem of identifying less frequent appearing behavior, interactive assessment is a promising approach for future studies. The methodology of interactive monitoring was developed by Myrtek (2004). Ebner-Priemer et al. (2013) introduced an algorithm for the analysis of the activity-affect relation. Diary signals can be triggered by an algorithm, developed for interactive monitoring, when a predefined activity threshold is surpassed. This method has the potential to quadruple the e-diary assessments during activity episodes compared to the method of this study (Ebner-Priemer et al., 2013).

Recently few authors claimed to analyze affective states not only pre and post but during exercise, because it is not yet determined whether during exercise affect or post exercise affect plays a greater role for future adherence to exercise and activity (Backhouse et al., 2007). Interactive monitoring offers the potential to meet the demand of exercise psychologists to measure affective states not only before and after exercise but also during exercise. Specific activities inducing positive affective states during an activity might be suitable for the promotion in daily life.

As sedentariness holds high risk factors for several diseases (Hamilton et al., 2004; Owen et al., 2010b; Warren et al., 2010) one of the main public health goals is getting sedentary people more active and achieve sustained active lifestyles. As current activity guidelines (Haskell et al., 2007) promote an increase in accumulated short bouts of PA in daily life, more studies examined the appearance of this behavior. Affect may influence one’s behavioral intention (Baumeister et al., 2007). Thus intensities, activities, and types of activity improving an individual’s affect may lead to future repetition of the behavior.

Based on the current literature one can assume that low intensity activities as often performed in daily life, can increase positive affect (Ekkekakis et al., 2000, 2008). Low intensity activities can increase affective states in sedentary inactive people because they experience them as less aversive than high intensity activities (Daley and Welch, 2003; Bixby and Lochbaum, 2006; Carels et al., 2006). In addition, they are easy to perform in daily life. Thus sedentary people might choose such activities to increase PA levels in daily life. The present study revealed a high inter- and intra-individual variability in affective responses to light intensity activity. This is in line with the claim of some authors to account for inter- and intra-individual variability and the dual mode theory (Backhouse et al., 2007). Due to the lack of active episodes no distinct conclusions can be drawn.

Randomized controlled trials increasing PA episodes and therefore data points to analyze may be a promising approach to get more insights into the daily life activity-affect relation of inactive people. To promote low intensity activity in daily life, for example, enhancing the accumulation of short bouts of activity or reducing longer periods of sedentary behavior by sedentary breaks may induce different affective reactions. Based on results of their study, Schwerdtfeger et al. 2008) assumed that obese people have the potential to increase energetic arousal through PA of daily life. This assumption should be taken into account when continuing research of the activity-affect association of inactive people in future projects based on the present study. Daily life activities having the potential to increase arousal (for example short walks) should be promoted by interventions and the effectiveness can be assessed by ambulatory assessment.

The present study has several limitations that should be discussed. The lack of moderate to vigorous activity episodes might reflect that assessing affect every 2 h over 2 days and relating it to the preceding 15 min before the PDA prompt may not be the method to choose in any sedentary sample. To get more active episodes, more days and more affect prompts have to be conducted. Another option to capture more active episode is looking for an active episode and relate it to the subsequent affect rating.

Clearly for the analysis of this ambulatory assessment study considering the association between PA and affect in daily life, multilevel analysis would be the common statistical method to use. It offers the potential to identify both within-subject and between-subject variability. Additionally, one can control confounding variables like contextual variables, habitual PA of subjects, and PA of the subsequent activity interval after the PDA prompt, etc., Due to the sample size, insufficient variance of the activity variables and the distribution (not normally distributed) of the valence and calmness variable in the present pilot study, we decided to calculate within-subject correlations instead of multilevel analyses. In addition, we wanted to take a special look on within-subject correlations.

However, there are some important aspects this study illustrates. The wide range of within-subject correlation coefficients seems to underline the responder-non-responder discussion or the consideration of the inter-individual affective reaction to PA in the activity-affect paradigm (Backhouse et al., 2007). It means that there might not only be one activity intensity, duration, and type of activity that enhances affective states. Every individual and its contextual situation might induce different reactions and different preferences of activity characteristics (as dose, duration, and type). Moreover, the low intraclass correlations in some participants illustrate, that some individuals’ affective response to PA differed from situation to situation. Both the inter- and intra-individual variability the present study revealed are in line with the concept of the dual mode theory. Interestingly, if participants were inactive, they showed high as well as low affective state scores, but lower affective states did not occur together with people being more active.

In conclusion, the present study attempted to address the potential of daily life activity to increase affective states of inactive people. The difficulty to capture active episodes of inactive people to identify which activities increase affect illustrated a general paradox. How can we assess which physical activities in daily life are useful for inactive people if they hardly perform any PA? Randomized controlled trials increasing PA in daily life, might solve this problem. However, inactive people probably do not like to be active, thus affective states might be negatively influenced by this aversion against activity. Future studies have to assess whether low intensity exercise and self-selected activities in daily life are less aversive to inactive people and thus useful to enhance activity in daily life.

## Conflict of Interest Statement

The authors declare that the research was conducted in the absence of any commercial or financial relationships that could be construed as a potential conflict of interest.
